# Genomic analysis of host gene responses to cerebral *Plasmodium falciparum* malaria

**DOI:** 10.1002/iid3.436

**Published:** 2021-05-04

**Authors:** Ke Li, Han Wang, Hong‐Feng Zhang, Xiao‐Xiao Zhao, Yong‐Ji Lai, Fang‐Fang Liu

**Affiliations:** ^1^ Department of Blood Transfusion, Tongji Hospital, Tongji Medical College Huazhong University of Science and Technology Wuhan China; ^2^ Department of Pharmacy, Chengdu Women's and Children's Central Hospital, School of Medicine University of Electronic Science and Technology of China Chengdu China; ^3^ Department of Pathology, The Central Hospital of Wuhan, Tongji Medical College Huazhong University of Science and Technology Wuhan China; ^4^ Department of Pharmacy, The Central Hospital of Wuhan, Tongji Medical College Huazhong University of Science and Technology Wuhan China

**Keywords:** cerebral malaria, coexpression networks, DEG, genomic analysis, *Plasmodium falciparum*

## Abstract

**Introduction:**

A vaccine for malaria is urgently required but no vaccine has yet shown satisfactory protective efficacy especially for *Plasmodium falciparum*. *P. falciparum* infection can progress to cerebral malaria (CM), a neurological syndrome with exceedingly high mortality. Designing effective *P. falciparum* vaccines require more understanding of the protective immune response while the host immune response to CM and the mechanisms are still elusive. Here, we aim to identify host gene responses to CM and host gene networks associated with CM pathogenesis.

**Methods:**

An innovative genomic analysis strategy, the weighted gene coexpression network analysis (WGCNA) combined with differential gene expression analysis, was used in this study. Data for analysis contain 93 whole blood samples, derived from two previous public transcriptome datasets.

**Results:**

This approach led to the identification of numerous differentially expressed human transcripts and dozens of coexpression gene modules. We further identified nine key genes, including MBP, SAMSN1, PSMF1, SLC39A8, EIF3B, SMPDL3A, FABP5, SPSB3, and SHARPIN, of which the last four genes were first identified to be related to CM in the present study.

**Conclusion:**

The results provided a comprehensive characterization of host gene expression profiles in CM and offered some new insight into malaria vaccine design. These identified key genes could be potential targets or immune modulators for novel therapeutic interventions of CM.

## Graphical abstract

The present study used an innovative genomic analysis strategy to explored gene coexpression networks associated with cerebral malaria (CM) pathogenesis and identified several key host genes. The findings will be of interest to host–parasite interaction study and would be of assistance to further investigate CM pathology.

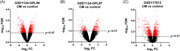

## 1 INTRODUCTION

Malaria is a devastating parasitic disease, leading to hundreds of thousands of pediatric deaths annually in sub‐Saharan Africa. The most common complication of malaria is severe malarial anemia (SMA) whereas the most severe is cerebral malaria (CM). CM is the major contributor to morbidity and mortality in the acute phase of severe malaria and the overall mortality rate for CM in children is 15%–25%.^1^ CM often has a poor prognosis and can lead to debilitating neurological impairments, epilepsy, blindness, deafness, or other irreversible sequelae, which may have an unclear effect on child development.^2^ Despite the considerable disease burden, the predictive diagnostics or treatments after onset are limited. Furthermore, the molecular bases and cellular processes resulting in this severe disease remain elusive.

The pathogenesis of CM is multifactorial and complicated. The pathologic hallmark of CM is the infected erythrocytes by *Plasmodium falciparum* in brain microvasculature, as shown in postmortem studies.^3^ So far, different studies have shown that the increased production of proinflammatory cytokines and chemokines plays an important role in CM development.^4^ Host immune responses in CM can lead to either protective or harmful outcomes.^5^ Seeking and a better understanding of those host genes/pathways in CM pathology will be useful in vaccine design. CM also has an effect on the expression of genes related to erythropoiesis and erythrocyte functions.^6^ However, the identification of host genes associated with CM pathology is still rudimentary and not fully investigated. Previous studies mostly tend to determine CM associated key gene through whether the gene is dramatically differentially expressed in CM (absolute value of the expression fold change [FC] higher than 1.5 or 2), but neglect the “fluctuation” of gene expression in disease development (from simple *P. falciparum* asymptomatic infection stage to CM status). For a gene, if its expression between different malaria phenotypes (from early stage to severe CM) shows an obvious correlation pattern, positive correlated or negative correlated, we may have reason to assume the potential importance of this gene in malaria pathogenesis. And if this gene is also differentially expressed in CM even with a relatively lower FC, it is very likely to play a vital role in CM pathogenesis and pathology.

Here, we applied a new genomic analysis strategy, weighted gene coexpression network analysis (WGCNA) combined with differential gene expression analysis. Raw data used in the present study were derived from two recently published malaria microarray datasets.^7^, ^8^ By utilizing this novel analysis strategy, we found numerous human transcripts related to CM pathogenesis and further identified nine key differentially expressed genes (DEGs). WGCNA is a systems biology method for describing the correlation patterns among genes and relating these gene sets (modules) to sample traits.^9^ Genes identified by WGCNA were more likely to be of functional importance. The nine identified genes showed strong associations with malaria phenotype/severity and also differentially expressed in CM versus healthy controls, this could be new biomarkers for the early diagnosis of CM as well as for the evaluation of effective therapeutic approaches in vivo in the future.

## 2 METHODS

### 2.1 Clinical information of raw data

Raw data for reanalysis comprising two array datasets (GSE1124 and GSE117613) were obtained from the Gene Expression Omnibus (GEO) database.^7^, ^8^ GSE1124 used two Affymetrix platforms, GPL96 and GPL97, which complement each other. GSE117613 used one Illumina platform GPL10558. Raw data were normalized via the robust multiarray average method. The GPL96 platform of the GSE1124 dataset includes 25 whole blood samples (healthy controls, *n* = 5; asymptomatic infection, *n* = 5; uncomplicated malaria, *n* = 5; SMA, *n* = 5; CM, *n* = 5). The GPL97 platform of the GSE1124 dataset includes 22 whole blood samples (healthy controls, *n* = 5; asymptomatic infection, *n* = 3; uncomplicated malaria, *n* = 5; SMA, *n* = 5; CM, *n* = 4). GSE117613 includes 46 whole blood samples (healthy controls, *n* = 12; SMA, *n* = 17; CM, *n* = 17). Overall, these datasets contain 93 whole blood samples. Clinical traits of samples were detailedly shown in Table S1.

#### 2.1.1 WGCNA and DEGs screening

For each array platform, we calculated the median absolute deviation and selected the top 5000 most variant genes to generate a weighted coexpression network. WGCNA was performed using “WGCNA” package^9^ in R (version 3.5.1). The weighted network analysis began with a matrix of the Pearson correlations between all gene pairs, then converts the correlation matrix into an adjacency matrix using a power function, so that results in an adjacency matrix—that is, the weighted coexpression network—is approximately scale‐free. The soft thresholding power (*β*) of the power function was determined based on the criterion of approximate scale‐free topology. Technically, the selected *β* value is the lowest power for which the scale‐free topology fit index reaches .90 (Figure S1). The minimal module size was set as 30. To define gene coexpression modules in the dataset, the adjacency matrix was used to calculate the topological overlap matrix (TOM), which shows the degree of overlap in shared neighbors between pairs of genes in the network. 1‐TOM was used as the dissimilarity measure for hierarchical clustering and module detection. Modules of clustered genes were then selected using the dynamic tree cut algorithm within WGCNA. To identify modules that are significantly associated with the measured clinical traits, expression profiles of each module were summarized by the module eigengene and the Pearson correlation between the module and the trait was calculated. The associations of individual genes with the malaria severity were quantified by gene significance (GS) value. The positive GS value represents a positive correlation with the malaria severity and vice versa. The R/Bioconductor (version 3.5.1) package “Limma” was used to screen DEGs. Limma was performed between CM and healthy controls respectively in the two datasets. Genes with a false discovery rate (FDR) of below 0.05 and an absolute FC higher than 1.5 or were considered differentially expressed. Limma results of the two datasets were merged in the end.

### 2.2 Key gene identification

Key genes were identified through the integration of WGCNA and Limma results. Since the GSE1124 dataset contains two complementary platforms, the analysis results of GSE1124 were the union of the two platforms but not the intersection of them. The results of GSE1124 and GSE117613 were then merged to obtain the overlapped genes. We then obtained key genes by further integrating the merged WGCNA results and the merged Limma results. An illustrative flowchart of the data analysis procedure was illustrated in Figure S2.

### 2.3 Statistics

Statistical analyses were performed using R software version 3.5.1. Pearson correlations between gene modules and malaria phenotype/severity were calculated within the WGCNA package in R. The FDR was determined by Benjamini–Hochberg method in the Limma package in R.

## 3 RESULTS

### 3.1 WGCNA identified gene coexpression modules associated with malaria severity

**Figure 1:**
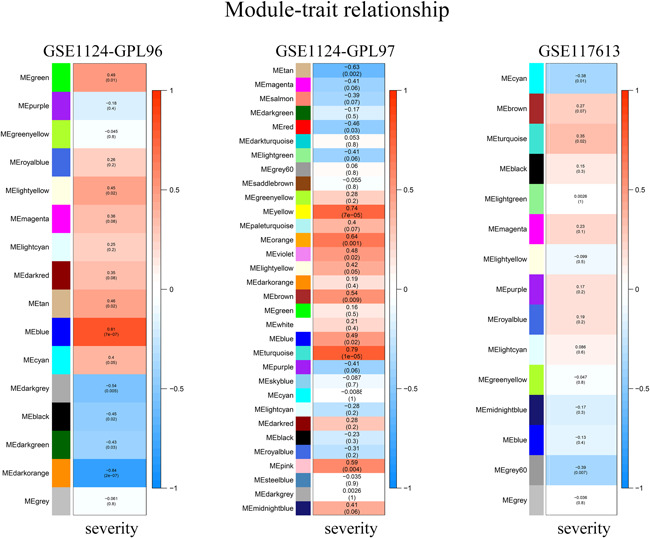
Gene coexpression modules related to cerebral malaria. Pearson correlation between modules and sample traits (malaria severity) in different platforms. Each row corresponds to a module identified on the left side by its color. The column corresponds to the trait (malaria severity). Numbers in cells report the correlations with the *p* values printed below the correlations in parentheses. Red is positively correlated and blue is negatively correlated

To identify coexpression modules related to malaria severity, we conducted WGCNA. Raw data used for WGCNA were obtained from the GEO database and consist of three platforms with 93 whole blood samples in total. For the GPL96 platform, WGCNA constructed 16 coexpression modules ranging in size from 47 to 1825 transcripts. Nine modules were found to be significantly correlated with malaria severity (Figure 1A). For the GPL97 platform, WGCNA clustered 32 modules, each comprising between 50 and 735 probes. Ten modules were highly related to malaria severity (Figure 1A). After integrating WGCNA results from the two platforms in the GSE1124 dataset, we obtained 6673 genes in all. Of the total, 3858 of the 6673 genes were positively correlated with malaria severity, the rest 2815 genes showed negative correlations. For the GPL10558 platform in the GSE117613 dataset, WGCNA yielded 15 modules, each containing 5–1173 transcripts. Three modules containing 1915 genes were significantly positively (963 genes) or negatively (952 genes) correlated with malaria severity (Figure 1). To visualize the weighted network more directly, we plotted heatmaps of the TOM among all genes in the analysis in each platform (Figure S3). The progressively darker red color represents higher overlap and higher correlation intensity. The WGCNA results of GSE1124 and GSE117613 were then merged together and 147 genes in total were found (Figure 3A and Table S2).

**Figure 2:**
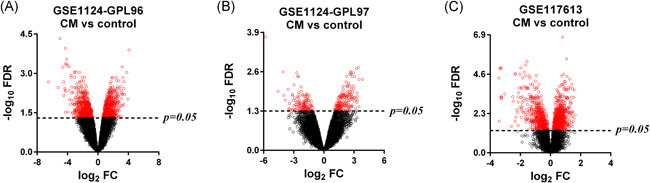
Venn diagram of the analysis results. (A) The Venn diagram depicts the number of genes identified by weighted gene coexpression network analysis (WGCNA) in the two datasets. (B) Venn diagrams show the upregulated and downregulated genes identified by Limma in the two datasets. (C) The Venn diagram depicts the integrated results, which is the overlap between WGCNA and Limma results. CM, cerebral malaria

### 3.2 Limma analysis identified DEGs in CM

**Figure 3:**
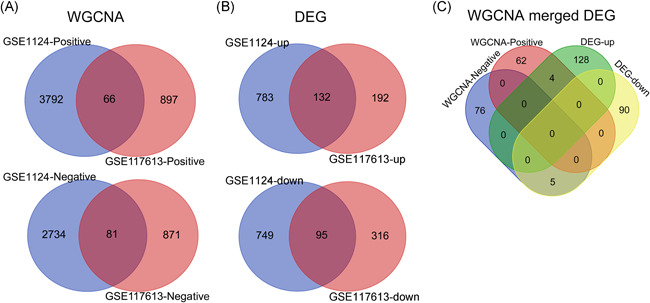
Volcano plot of differentially expressed genes (DEGs) in three platforms. The *X*‐axis represents log_2_FC and the *Y*‐axis indicates the negative log_10_FDR. Each dot (circle) represents one probe that had a detectable expression in both groups. Red dots represent probes that are significantly expressed in cerebral malaria compared with healthy control. FC, fold change; FDR, false discovery rate

To identify DEGs between CM and control, we applied the Limma approach. The cutoff criteria were set as FDR < 0.05 and FC ≥ 1.5. The Limma method identified 1583, 210, and 735 DEGs in the GPL96, GPL97, and GPL10558 platforms for CM versus control, respectively (Figure 2A–C). After combining the GPL96 and GPL97 platforms, 1579 DEGs with 844 upregulated and 915 downregulated were found in the GSE1124 dataset. Among all these identified DEGs, 227 were found shared between GSE1124 and GSE117613, including 95 upregulated and 132 downregulated DEGs (Figure 3B and Table S3).

### 3.3 Key gene identification

**Figure 4:**
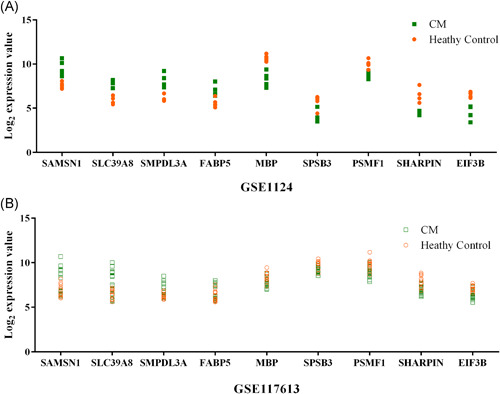
Dot plots illustrating log_2_gene expression values of nine key genes across cerebral malaria (CM) and healthy control groups. The dots show individual patient data and are color‐coded to reflect different groups

To identify key genes associated with CM, we integrated WGCNA and Limma results. Nine common genes were found shared between WGCNA and Limma (Figure 3C and Table 1). Within the nine genes, five genes (MBP, SAMSN1, PSMF1, SLC39A8, and EIF3B) were previously found to be involved in malaria,^10^, ^11^, ^12^, ^13^, ^14^ and the remaining four genes (SMPDL3A, FABP5, SPSB3, and SHARPIN) were identified for the first time in the current study. SAMSN1, SLC39A8, SMPDL3A, and FABP5 were positively correlated with malaria phenotype/severity and showed an upregulation in the CM group compared with healthy controls, while MBP, SPSB3, PSMF1, SHARPIN, EIF3B were negatively correlated with malaria severity and downregulated in the DEG (Figure 3C and Table 1). The nine key genes have different GS and FC values in the two datasets. In the GSE1124 dataset, the most downregulated was MBP at 0.2‐fold and the most upregulated gene was SMPDL3A at 3.9‐fold, while the most relevant genes with CM were SAMSN1 (positive correlation, GS = 0.79019052) and EIF3B (negative correlation, GS = −0.74050498). In the GSE117613 dataset, the most DEGs were SHARPIN (log_2_FC = −0.968490676) and SAMSN1 (log_2_FC = 1.492716272), and the most relevant genes were SLC39A8 (positive correlation, GS = 0.330973954) and SPSB3 (negative correlation, GS = −0.395300297). Figure 4 shows the relative expression levels of the nine key genes between CM and healthy control samples in the two datasets.

**Table 1:** Nine key DEGs highly correlated with CM pathology

Gene symbol	Entrez ID	Location	GSE1124				GSE117613				Ref.
			GS	p.GS	log_2_FC	FDR	GS	p.GS	log_2_FC	FDR	
SAMSN1	64092	21q11	0.790191	2.62E−06	1.951153	0.014014	0.3246556	0.02771247	1.492716	0.000174	Muehlenbachs et al.
SLC39A8	64116	4q24	0.711455	6.68E−05	1.857339	0.005491	0.330974	0.02465693	1.38601	0.003471	Idaghdour et al.
SMPDL3A	10924	6q22.31	0.708142	7.48E−05	1.968451	0.012602	0.1756297	0.24300915	1.297318	0.000524	–
FABP5	2171	8q21.13	0.620998	0.000924	1.360967	0.036907	0.2897648	0.05078081	0.731826	0.001235	–
MBP	4155	18q23	−0.12736	0.544074	−2.37729	0.006302	−0.2242893	0.13400206	−0.86847	0.007388	Leitner et al.
SPSB3	90864	16p13.3	−0.58106	0.00232	−1.65651	0.032354	−0.3953003	0.00654806	−0.62352	0.010131	–
PSMF1	9491	20p13	−0.62173	0.000908	−1.11549	0.036333	−0.3714011	0.01104537	−0.91604	0.009882	Sobota et al.
SHARPIN	81858	8q24.3	−0.68517	0.000157	−2.0102	0.008372	−0.0732082	0.62873543	−0.96849	0.003727	–
EIF3B	8662	7p22.3	−0.7405	8.12E−05	−2.05801	0.034577	−0.3089987	0.0366634	−0.69376	0.00204	Tuteja et al.

*Note*: GS and p.GS were generated from WGCNA; log_2_FC and FDR were calculated in Limma.

Abbreviations: CM, cerebral malaria; FDR, false discovery rate; GS, gene significance; log_2_FC, log_2_(fold change of CM vs. control); WGCNA, weighted gene coexpression network analysis.

## 4 DISCUSSION

CM is the most lethal complication among all malaria syndromes. In the present study, we used WGCNA combined DEG analysis to identify key genes in CM. WGCNA is a useful approach to link clustered genes to phenotypic traits. However, not all of those genes identified by WGCNA were differentially expressed between CM and controls. Therefore, we further applied DEG analysis (Limma method) to improve WGCNA results. In total, we detected 227 genes that were both differentially regulated in CM and associated with disease severity. Nine key genes (MBP, SAMSN1, PSMF1, SLC39A8, EIF3B, SMPDL3A, FABP5, SPSB3, and SHARPIN) were further identified from the analysis results.

Among nine key genes, four genes (SAMSN1, SLC39A8, SMPDL3A, and FABP5) were positively correlated with malaria severity and upregulated in CM. SAMSN1 and SLC39A8 were previously found upregulated in malaria.^10^, ^12^ SAMSN1, also known as HACS1, is mainly expressed in hematopoietic and endothelial cells, usually acts as an immunoinhibitory factor and modulates B‐cell activation and differentiation.^15^ B‐cell has been traditionally considered an antibody‐producing cell and plays an important role in the regulation of immune response. In malaria, plenty of parasitic antigens are expressed in each stage of the parasite life cycle.^16^ The parasite exposure results in B‐cell activation and differentiation into Plasmodium‐specific memory B‐cell.^17^ To escape the host humoral responses, Plasmodium parasites can disturb the function of B‐cell.^18^ Thus, the increased SAMSN1 is possibly due to the activation of B‐cell in malaria infection. Another key gene, SLC39A8, has been reported associated with malaria susceptibility.^10^ SLC39A8 encodes a zinc transporter protein ZIP8. Previous studies showed that ZIP8 was markedly upregulated upon T‐cell activation, especially in the presence of low concentrations of zinc.^19^ In fact, the activation of T‐cell, particularly CD4 + T cell subset, is the common immune process during malaria infection and may affect the effectiveness of humoral responses. The elevated expression of SLC39A8 probably reflected T‐cell activation in CM. The identified key gene SMPDL3A was the most significant DEG in the GSE1124 dataset among the nine key genes. SMPDL3A is a recently identified phosphodiesterase and ubiquitously expressed in the human body.^20^ SMPDL3A is one of three enzymes of the sphingomyelinase (SMase) family, the remaining two of which are SMPDL3B and SMPD1. Enhanced eryptosis has been observed in malaria.^21^ Interestingly, it has been reported recently that some compounds, such as amitriptyline and flufenamic acid, could suppress eryptosis by inhibiting sphingomyelinase.^22^ Therefore, the increased SMPDL3A was possibly an indicator of eryptosis in malaria.

Conversely, the other five key genes (MBP, SPSB3, PSMF1, SHARPIN, and EIF3B) were negatively correlated with malaria severity and decreased in CM. The downregulation of MBP in CM has also been found in recent research.^11^ MBP is a multifunctional protein mainly expressed in the brain and thyroid. Actually, MBP is a major constituent of the myelin sheath of oligodendrocytes and Schwann cells. Axonal and myelin damage were commonly present in CM patients.^23^ Studies in the CM mouse model have also shown that the level of MBP declines by approximately 80% in contrast to the control.^11^ SPSB3 was a newly identified gene involved in CM pathology in the present study. SPSB3 is a SOCS box protein belonging to the sp1A/ryanodine receptor (SPRY) family, which has been found to be involved in stress response and cytokine signaling.^24^ More recently, studies have shown SPSB3 could be served as a novel E3 ubiquitin ligase.^25^ Interestingly, the inhibition of ubiquitin E3 ligase has been found to have antimalarial effects.^26^ Hence, we speculated that the reduction of SPSB3 could be a compensatory process during the development of CM. Besides, the other identified key gene, PSMF1 and EIF3B, have also been reported associated with malaria in previous studies.^13^, ^14^

The present study was based on a public dataset derived from a previous study.^7^, ^8^ The raw data were available at the GEO database. There were also important limitations to this study. First, despite the strong associations between the identified genes and disease severity, we could not confirm the causality. Second, although the raw data used in this study were comprised of large numbers of samples, we lacked enough resources to validate our findings. Notwithstanding the limitations, the present results demonstrated that CM has different blood transcriptional signatures in contrast to uncomplicated malaria and SMA. The results offer valuable insights into the potential molecular bases in CM.

## CONFLICT OF INTERESTS

The authors declare that there are no conflict of interests.

## AUTHOR CONTRIBUTIONS

*Conceptualization and methodology*: Fang‐Fang Liu and Yong‐Ji Lai. *Data analysis*: Ke Li, Han Wang, Hong‐Feng Zhang, and Xiao‐Xiao Zhao. *Manuscript writing*: Ke Li, Fang‐Fang Liu, and Yong‐Ji Lai.

## Supporting information

Supporting information.

## Data Availability

The data that support the findings of this study are openly available in The Gene Expression Omnibus (GEO) database at: https://www.ncbi.nlm.nih.gov/geo/query/acc.cgi?acc=GSE117613, reference number GSE117613; https://www.ncbi.nlm.nih.gov/geo/query/acc.cgi?acc=GSE1124, reference number GSE1124.
