# Supplementary material for: Genomic analysis of host gene responses to cerebral Plasmodium falciparum malaria
Source: Immun Inflamm Dis. 2021 May 4;9(3):819–26. doi: 10.1002/iid3.436 (PMC8342194; doi:10.1002/iid3.436)

Supplementary Data

Supplementary Figure legends

Supplementary Figure 1. Analysis of network topology for various soft-thresholding powers. **(A-C).** The left panel shows the scale-free fit index (y-axis) as a function of the soft-thresholding power (x-axis). The right panel displays the mean connectivity (degree, y-axis) as a function of the soft-thresholding power (x-axis).

Supplementary Figure 2. Overview of data analysis procedure. This flowchart presents a brief overview of the main steps of the microarray the data analysis procedure in the present study.

Supplementary Figure 3. Heatmap of TOM among transcripts in GSE1124-GPL96 (A), GSE1124-GPL97 (B), and GSE117613-GPL10558 (C) platforms. Light color represents low overlap and the progressively darker red color represents higher overlap. Rows and columns were symmetric and represent genes, which are sorted by WGCNA algorithm. The different shades of color signify the strength of the connections between the nodes (from white signifying not significantly correlated to red signifying highly significantly correlated). The hierarchical clustering and the TOM indicate highly interconnected subsets of genes (modules). Blocks of darker colors along the diagonal are the modules. Modules identified are also colored along both column and row and are boxed. TOM, Topological Overlap Matrix.

Supplementary Table 1. Clinical traits of samples in GSE117613 and GSE1124.

Accession	Dataset	Platform	DIESEASE STATUS	SEX	AGE
GSM3305150	GSE117613	GPL10558	community children withou	Male	15.5
GSM3305151	GSE117613	GPL10558	severe malaral anemia	Male	23
GSM3305152	GSE117613	GPL10558	severe malaral anemia	Male	12
GSM3305153	GSE117613	GPL10558	severe malaral anemia	Male	19
GSM3305154	GSE117613	GPL10558	severe malaral anemia	Female	10
GSM3305155	GSE117613	GPL10558	severe malaral anemia	Female	14
GSM3305156	GSE117613	GPL10558	cerebral milaria	Male	12
GSM3305157	GSE117613	GPL10558	severe malaral anemia	Male	16
GSM3305158	GSE117613	GPL10558	cerebral milaria	Female	12
GSM3305159	GSE117613	GPL10558	severe malaral anemia	Female	14
GSM3305160	GSE117613	GPL10558	severe malaral anemia	Female	11
GSM3305161	GSE117613	GPL10558	severe malaral anemia	Female	9.5
GSM3305162	GSE117613	GPL10558	severe malaral anemia	Male	9.8
GSM3305163	GSE117613	GPL10558	severe malaral anemia	Female	11
GSM3305164	GSE117613	GPL10558	cerebral milaria	Female	12
GSM3305165	GSE117613	GPL10558	community children withou	Male	14
GSM3305166	GSE117613	GPL10558	community children withou	Female	11
GSM3305167	GSE117613	GPL10558	cerebral milaria	Male	11
GSM3305168	GSE117613	GPL10558	cerebral milaria	Male	13
GSM3305169	GSE117613	GPL10558	cerebral milaria	Female	15
GSM3305170	GSE117613	GPL10558	cerebral milaria	Male	12.2
GSM3305171	GSE117613	GPL10558	cerebral milaria	Female	14
GSM3305172	GSE117613	GPL10558	severe malaral anemia	Male	20.6
GSM3305173	GSE117613	GPL10558	cerebral milaria	Male	16
GSM3305174	GSE117613	GPL10558	community children withou	Female	10.3
GSM3305175	GSE117613	GPL10558	severe malaral anemia	Male	13.6
GSM3305176	GSE117613	GPL10558	cerebral milaria	Male	11.5
GSM3305177	GSE117613	GPL10558	severe malaral anemia	Male	12.2
GSM3305178	GSE117613	GPL10558	community children withou	Female	35.4
GSM3305179	GSE117613	GPL10558	cerebral milaria	Male	18
GSM3305180	GSE117613	GPL10558	severe malaral anemia	Male	9
GSM3305181	GSE117613	GPL10558	cerebral milaria	Female	13
GSM3305182	GSE117613	GPL10558	severe malaral anemia	Female	13.2
GSM3305183	GSE117613	GPL10558	severe malaral anemia	Male	8
GSM3305184	GSE117613	GPL10558	cerebral milaria	Male	10
GSM3305185	GSE117613	GPL10558	community children withou	Female	11
GSM3305186	GSE117613	GPL10558	community children withou	Female	19.8
GSM3305187	GSE117613	GPL10558	community children withou	Male	10
GSM3305188	GSE117613	GPL10558	community children withou	Female	16
GSM3305189	GSE117613	GPL10558	cerebral milaria	Male	12
GSM3305190	GSE117613	GPL10558	cerebral milaria	Male	10
GSM3305191	GSE117613	GPL10558	cerebral milaria	Male	12.5
GSM3305192	GSE117613	GPL10558	community children withou	Female	14.3
GSM3305193	GSE117613	GPL10558	community children withou	Female	16.2
GSM3305194	GSE117613	GPL10558	cerebral milaria	Female	7.8
GSM3305195	GSE117613	GPL10558	community children withou	Male	15.8
GSM711619	GSE1124	GPL96	healthy	-	-
GSM711620	GSE1124	GPL96	healthy	-	-
GSM711621	GSE1124	GPL96	healthy	-	-
GSM711622	GSE1124	GPL96	healthy	-	-
GSM711623	GSE1124	GPL96	healthy	-	-

GSM711624	GSE1124	GPL96	with asymptomatic Plasmoc	-	-
GSM711625	GSE1124	GPL96	with asymptomatic Plasmoc	-	-
GSM711626	GSE1124	GPL96	with asymptomatic Plasmoc	-	-
GSM711627	GSE1124	GPL96	with asymptomatic Plasmoc	-	-
GSM711628	GSE1124	GPL96	with asymptomatic Plasmoc	-	-
GSM711629	GSE1124	GPL96	with uncomplicated malaria	-	-
GSM711630	GSE1124	GPL96	with uncomplicated malaria	-	-
GSM711631	GSE1124	GPL96	with uncomplicated malaria	-	-
GSM711632	GSE1124	GPL96	with uncomplicated malaria	-	-
GSM711633	GSE1124	GPL96	with uncomplicated malaria	-	-
GSM711634	GSE1124	GPL96	with malaria associated witl	-	-
GSM711635	GSE1124	GPL96	with malaria associated witl	-	-
GSM711636	GSE1124	GPL96	with malaria associated witl	-	-
GSM711637	GSE1124	GPL96	with malaria associated witl	-	-
GSM711638	GSE1124	GPL96	with malaria associated witl	-	-
GSM711639	GSE1124	GPL96	with cerebral malaria	-	-
GSM711640	GSE1124	GPL96	with cerebral malaria	-	-
GSM711641	GSE1124	GPL96	with cerebral malaria	-	-
GSM711642	GSE1124	GPL96	with cerebral malaria	-	-
GSM711643	GSE1124	GPL96	with cerebral malaria	-	-
GSM711644	GSE1124	GPL97	healthy	-	-
GSM711645	GSE1124	GPL97	healthy	-	-
GSM711646	GSE1124	GPL97	healthy	-	-
GSM711647	GSE1124	GPL97	healthy	-	-
GSM711648	GSE1124	GPL97	healthy	-	-
GSM711649	GSE1124	GPL97	with asymptomatic Plasmoc	-	-
GSM711650	GSE1124	GPL97	with asymptomatic Plasmoc	-	-
GSM711651	GSE1124	GPL97	with asymptomatic Plasmoc	-	-
GSM711652	GSE1124	GPL97	with uncomplicated malaria	-	-
GSM711653	GSE1124	GPL97	with uncomplicated malaria	-	-
GSM711654	GSE1124	GPL97	with uncomplicated malaria	-	-
GSM711655	GSE1124	GPL97	with uncomplicated malaria	-	-
GSM711656	GSE1124	GPL97	with uncomplicated malaria	-	-
GSM711657	GSE1124	GPL97	with malaria associated witl	-	-
GSM711658	GSE1124	GPL97	with malaria associated witl	-	-
GSM711659	GSE1124	GPL97	with malaria associated witl	-	-
GSM711660	GSE1124	GPL97	with malaria associated witl	-	-
GSM711661	GSE1124	GPL97	with malaria associated witl	-	-
GSM711662	GSE1124	GPL97	with cerebral malaria	-	-
GSM711663	GSE1124	GPL97	with cerebral malaria	-	-
GSM711664	GSE1124	GPL97	with cerebral malaria	-	-
GSM711665	GSE1124	GPL97	with cerebral malaria	-	-

Supplementary Table 2. 147 genes identified both in GSE1124 and GSE117613 by WGCNA. GS: Gene Significance.

Genesymbol	GeneID	Location	GSE1124-GPL96		GSE1124-GPL97		GSE117613-GPL10558	
			GS	*p*.GS	GS	*p*.GS	GS	*p*.GS
U2AF1	7307	21q22.3	-0.383094	0.058725	0.690044	0.000380	0.438662	0.002292
LARP4	113251	12q13.12	N/A	N/A	0.277220	0.211652	0.426510	0.003119
MRPL30	51263	2q11.2	N/A	N/A	0.079905	0.723733	0.423994	0.003320
PRDM1	639	6q21	0.235651	0.256796	N/A	N/A	0.401155	0.005728
ZFP36L1	677	14q24.1	0.282019	0.171997	N/A	N/A	0.385199	0.008205
SLC2A14	144195	12p13.31	0.771849	0.000006	N/A	N/A	0.376950	0.009815
FNDC3A	22862	13q14.2	0.385836	0.056789	N/A	N/A	0.367118	0.012083
MRPL47	57129	3q26.33	N/A	N/A	0.492826	0.019788	0.366380	0.012270
SEC24A	10802	5q31.1	N/A	N/A	0.039670	0.860864	0.363149	0.013118
TSEN15	116461	1q25	N/A	N/A	0.512666	0.014701	0.355776	0.015243
HOXC6	3223	12q13.3	0.324382	0.113650	N/A	N/A	0.353004	0.016114
ATP1B3	483	3q23	N/A	N/A	0.607064	0.002736	0.348795	0.017517
G3BP1	10146	5q33.1	0.048413	0.818243	N/A	N/A	0.348519	0.017612
ACER2	340485	9p22.1	N/A	N/A	0.388336	0.074101	0.347932	0.017817
HSPD1	3329	2q33.1	0.536102	0.005739	-0.221112	0.322722	0.345644	0.018634
NXT2	55916	Xq23	0.478788	0.015465	N/A	N/A	0.342732	0.019719
PHF3	23469	6q12	0.086032	0.682617	N/A	N/A	0.339708	0.020901
MTHFD2	10797	2p13.1	N/A	N/A	0.355860	0.104073	0.331414	0.024455
INPP5F	22876	10q26.11	N/A	N/A	0.480885	0.023477	0.331030	0.024631
BCL2A1	597	15q24.3	0.731758	0.000032	N/A	N/A	0.330407	0.024919
FBXO6	26270	1p36.22	N/A	N/A	0.498944	0.018088	0.326212	0.026932
FAM162A	26355	3q21.1	0.015356	0.941923	N/A	N/A	0.319247	0.030572
SKAP2	8935	7p15.2	0.447561	0.024872	0.554535	0.007399	0.309641	0.036254
MS4A4A	51338	11q12	0.673428	0.000225	0.683217	0.000457	0.304123	0.039893
B3GAT1	27087	11q25	0.059808	0.776424	N/A	N/A	0.301569	0.041676
SLC39A8	64116	4q24	0.711455	0.000067	0.264121	0.234928	0.300814	0.042216
BOLA2	552900	16p11.2	N/A	N/A	0.207898	0.353196	0.293985	0.047357
TSC22D3	1831	Xq22.3	0.201598	0.333867	N/A	N/A	0.287470	0.052725
ANP32A	8125	15q23	0.530872	0.006327	N/A	N/A	0.282591	0.057058
B4GALT4	8702	3q13.3	0.435354	0.029619	N/A	N/A	0.280626	0.058882
AGFG1	3267	2q36.3	0.566030	0.003184	N/A	N/A	0.270952	0.068554
DBI	1622	2q12-q21	0.360775	0.076437	N/A	N/A	0.263277	0.077090
FABP5	2171	8q21.13	0.620998	0.000924	N/A	N/A	0.260238	0.080693
MAZ	4150	16p11.2	0.173503	0.406866	N/A	N/A	0.251109	0.092312
MCL1	4170	1q21	0.228233	0.272508	N/A	N/A	0.243740	0.102605
SAMSN1	64092	21q11	0.790191	0.000003	N/A	N/A	0.239657	0.108676
HK2	3099	2p13	-0.194996	0.350265	0.153579	0.495008	0.227892	0.127703
CLIC4	25932	1p36.11	0.546979	0.004662	N/A	N/A	0.221846	0.138404
RIN2	54453	20p11.22	0.541747	0.005156	0.003355	0.988177	0.218451	0.144700
FGD2	221472	6p21.2	N/A	N/A	0.012337	0.956545	0.216125	0.149133
BCAT1	586	12p12.1	0.444509	0.025997	0.822349	0.000003	0.191936	0.201297
NEK2	4751	1q32.3	0.515735	0.008320	N/A	N/A	0.185818	0.216312
FASTK	10922	7q35	0.010681	0.959586	N/A	N/A	0.179169	0.233494
ERGIC2	51290	12p11.22	N/A	N/A	0.127125	0.572918	0.179117	0.233632
EIF4E	1977	4q23	N/A	N/A	0.306617	0.165163	0.172301	0.252198
SMPDL3A	10924	6q22.31	0.708142	0.000075	N/A	N/A	0.169191	0.260989

THBS1	7057	15q15	0.635488	0.000641	N/A	N/A	0.159562	0.289498
AIF1	199	6p21.3	0.384325	0.057850	N/A	N/A	0.157902	0.294611
PTGS2	5743	1q25.2	0.183315	0.380424	N/A	N/A	0.141596	0.347905
SYNJ1	8867	21q22.2	N/A	N/A	0.115878	0.607583	0.136337	0.366282
EPHA10	284656	1p34.3	N/A	N/A	0.315677	0.152395	0.135733	0.368426
NUCKS1	64710	1q32.1	N/A	N/A	0.510459	0.015208	0.123538	0.413382
SAR1A	56681	10q22.1	0.073657	0.726410	N/A	N/A	0.123185	0.414729
SAP30	8819	4q34.1	0.479698	0.015243	N/A	N/A	0.118853	0.431453
APOL2	23780	22q12	0.289606	0.160259	N/A	N/A	0.116497	0.440704
CCND2	894	12p13	0.246708	0.234489	N/A	N/A	0.111307	0.461466
ZBP1	81030	20q13.31	N/A	N/A	0.184088	0.412164	0.103868	0.492119
TXNL1	9352	18q21.31	N/A	N/A	0.090139	0.689950	0.101064	0.503943
MFGE8	4240	15q25	0.190625	0.361381	N/A	N/A	0.099286	0.511511
MCTP1	79772	5q15	N/A	N/A	0.749609	0.000059	0.080263	0.595940
SNRPN	6638	15q11.2	N/A	N/A	0.143688	0.523512	0.073146	0.629030
PRRG4	79056	11p13	0.481218	0.014877	N/A	N/A	0.060329	0.690430
TSC22D2	9819	3q25.1	0.333542	0.103233	N/A	N/A	0.059632	0.693827
MET	4233	7q31	0.495796	0.011722	N/A	N/A	0.034673	0.819053
GZMB	3002	14q11.2	0.541546	0.005176	N/A	N/A	0.033890	0.823077
WSB1	26118	17q11.1	0.442513	0.026754	N/A	N/A	-0.014547	0.923558
MPHOSPH10	10199	2p13.3	-0.159683	0.445792	N/A	N/A	-0.038283	0.800582
ZADH2	284273	18q22.3	N/A	N/A	-0.288244	0.193298	-0.050926	0.736789
CALM3	808	19q13.2	-0.015237	0.942371	-0.267160	0.229386	-0.065266	0.666519
GNPTAB	79158	12q23.2	N/A	N/A	-0.231509	0.299896	-0.065516	0.665314
SHARPIN	81858	8q24.3	-0.685175	0.000157	N/A	N/A	-0.073208	0.628735
HBZ	3050	16p13.3	-0.806240	0.000001	N/A	N/A	-0.076850	0.611712
NAPSB	256236	19q13.33	N/A	N/A	-0.483029	0.022777	-0.078133	0.605762
SP110	3431	2q37.1	-0.681991	0.000174	N/A	N/A	-0.092047	0.542918
CORO2A	7464	9q22.3	N/A	N/A	-0.246729	0.268323	-0.095855	0.526283
PTPRE	5791	10q26	N/A	N/A	-0.351619	0.108560	-0.106178	0.482491
DGUOK	1716	2p13	-0.175969	0.400127	N/A	N/A	-0.134982	0.371108
CITED2	10370	6q23.3	-0.001062	0.995979	N/A	N/A	-0.137967	0.360523
CD52	1043	1p36	-0.448353	0.024586	N/A	N/A	-0.138507	0.358628
WDFY2	115825	13q14.3	N/A	N/A	-0.465777	0.028911	-0.152297	0.312300
DPM3	54344	1q22	-0.476726	0.015979	N/A	N/A	-0.159853	0.288607
CST3	1471	20p11.21	-0.638717	0.000590	N/A	N/A	-0.160911	0.285387
SDF4	51150	1p36.33	-0.371850	0.067200	N/A	N/A	-0.163721	0.276946
DIP2A	23181	21q22.3	-0.228806	0.271274	N/A	N/A	-0.167743	0.265153
ZNF23	7571	16q22.2	-0.039650	0.850741	N/A	N/A	-0.171478	0.254503
TMEM158	25907	3p21.3	-0.032170	0.878670	N/A	N/A	-0.171684	0.253926
ALG13	79868	Xq23	-0.460697	0.020470	N/A	N/A	-0.176615	0.240333
PQLC1	80148	18q23	N/A	N/A	-0.226755	0.310205	-0.190985	0.203581
ZBTB44	29068	11q24.3	-0.657546	0.000355	N/A	N/A	-0.193716	0.197069
ATP5E	514	20q13.32	N/A	N/A	-0.348962	0.111443	-0.198986	0.184922
MARK2	2011	11q13.1	-0.043784	0.835377	N/A	N/A	-0.199097	0.184673
MPPE1	65258	18p11.21	-0.387134	0.055891	N/A	N/A	-0.199122	0.184616
CD2AP	23607	6p12	N/A	N/A	-0.260664	0.241338	-0.208469	0.164431
MBP	4155	18q23	-0.127357	0.544074	N/A	N/A	-0.224289	0.134002
ITM2B	9445	13q14.3	-0.675873	0.000209	N/A	N/A	-0.227273	0.128769
STK4	6789	20q11.2	-0.295028	0.152225	-0.579007	0.004750	-0.236406	0.113702
SAFB2	9667	19p13.3	-0.178978	0.391989	N/A	N/A	-0.239037	0.109621
HIST1H3F	8968	6p22.2	-0.040614	0.847153	N/A	N/A	-0.242784	0.104002

CMAS	55907	12p12.1	N/A	N/A	-0.210730	0.346528	-0.247524	0.097214
ARF1	375	1q42	N/A	N/A	-0.650322	0.001050	-0.253106	0.089666
ACTA2	59	10q23.3	0.449925	0.024028	-0.303908	0.169123	-0.257606	0.083918
FCRL5	83416	1q21	N/A	N/A	-0.342939	0.118182	-0.259259	0.081881
PRDX2	7001	19p13.2	-0.551268	0.004287	N/A	N/A	-0.268276	0.071440
BACE2	25825	21q22.3	N/A	N/A	-0.314235	0.154379	-0.269292	0.070333
FIP1L1	81608	4q12	-0.520693	0.007617	N/A	N/A	-0.272258	0.067179
ZNF653	115950	19p13.2	N/A	N/A	-0.034182	0.879967	-0.273730	0.065656
ID2	3398	2p25	-0.498441	0.011213	N/A	N/A	-0.289897	0.050671
XPO7	23039	8p21	0.377161	0.063087	-0.469371	0.027536	-0.301550	0.041689
EIF3B	8662	7p22.3	N/A	N/A	-0.740505	0.000081	-0.308999	0.036663
CHD2	1106	15q26	N/A	N/A	-0.475073	0.025461	-0.312447	0.034512
TSPAN5	10098	4q23	-0.565534	0.003217	-0.049469	0.826946	-0.318255	0.031123
STK40	83931	1p34.3	N/A	N/A	-0.329876	0.133803	-0.321986	0.029095
UBAP2L	9898	1q21.3	-0.288525	0.161897	N/A	N/A	-0.324343	0.027872
GSDMB	55876	17q12	-0.354402	0.082172	N/A	N/A	-0.330971	0.024659
SF3A2	8175	19p13.3	-0.185130	0.375643	N/A	N/A	-0.332279	0.024062
BCL2L1	598	20q11.21	0.298032	0.147900	-0.415355	0.054562	-0.339341	0.021049
ASMTL	8623	Xp22.3	-0.564424	0.003291	N/A	N/A	-0.341555	0.020172
MAT2B	27430	5q34	N/A	N/A	-0.012205	0.957008	-0.354882	0.015519
TRIM10	10107	6p21.3	-0.036934	0.860863	N/A	N/A	-0.355018	0.015477
PCM1	5108	8p22	N/A	N/A	-0.177116	0.430392	-0.357187	0.014815
HPS1	3257	10q23.1	N/A	N/A	-0.199864	0.372515	-0.370066	0.011360
PSMF1	9491	20p13	-0.621725	0.000908	N/A	N/A	-0.371401	0.011045
CYSLTR1	10800	Xq13.2	N/A	N/A	-0.733774	0.000102	-0.375750	0.010071
TCEB2	6923	16p12.3	-0.181551	0.385102	N/A	N/A	-0.382081	0.008785
LZTFL1	54585	3p21.3	N/A	N/A	-0.026155	0.908020	-0.384340	0.008361
CAT	847	11p13	-0.761547	0.000010	N/A	N/A	-0.385167	0.008211
PCGF5	84333	10q23.32	N/A	N/A	-0.070610	0.754853	-0.385568	0.008139
RANBP10	57610	16q22.1	-0.238147	0.251646	0.237724	0.286738	-0.389120	0.007523
DDX24	57062	14q32	-0.754003	0.000013	N/A	N/A	-0.395153	0.006570
SPSB3	90864	16p13.3	-0.581056	0.002320	N/A	N/A	-0.395300	0.006548
CD3G	917	11q23	-0.723999	0.000043	N/A	N/A	-0.396715	0.006341
PPP2R5B	5526	11q12	-0.354679	0.081916	N/A	N/A	-0.412516	0.004387
NFIX	4784	19p13.3	-0.475501	0.016291	N/A	N/A	-0.419819	0.003678
YIPF6	286451	Xq12	-0.254165	0.220190	N/A	N/A	-0.428388	0.002976
ILF3	3609	19p13.2	-0.040378	0.848032	N/A	N/A	-0.432220	0.002703
FAM20B	9917	1q25	-0.222245	0.285629	N/A	N/A	-0.434850	0.002528
C18orf8	29919	18q11.2	N/A	N/A	-0.686713	0.000416	-0.447541	0.001817
TM7SF2	7108	11q13	-0.063687	0.762317	N/A	N/A	-0.467058	0.001067
GSK3B	2932	3q13.3	N/A	N/A	-0.019456	0.931516	-0.474269	0.000869
ABCC4	10257	13q32	N/A	N/A	-0.082554	0.714937	-0.477334	0.000796
WDR45	11152	Xp11.23	-0.592484	0.001804	N/A	N/A	-0.477550	0.000791
POLD4	57804	11q13	-0.415441	0.038898	N/A	N/A	-0.479039	0.000757
ARHGEF12	23365	11q23.3	0.176797	0.397878	-0.254420	0.253202	-0.482264	0.000689
ZER1	10444	9q34.11	-0.310006	0.131526	N/A	N/A	-0.484950	0.000636
RORA	6095	15q22.2	N/A	N/A	-0.496438	0.018770	-0.543893	0.000094
KPNA6	23633	1p35.1	-0.111930	0.594258	N/A	N/A	-0.551976	0.000070
WDR48	57599	3p21.33	-0.422109	0.035565	N/A	N/A	-0.577074	0.000027

Supplementary Table 3. 227 genes identified both in GSE1124 and GSE117613 by Limma. log_2FC: log_2(Fold Change); FDR: false discovery rate.

Genesymbol	GSE1124-GPL96		GSE1124-GPL97		GSE117613-GPL10558	
	log_2FC	FDR	log_2FC	FDR	log_2FC	FDR
FLT3LG	-3.310960	0.001408	N/A	N/A	-1.316600	0.000955
RBL2	-3.235619	0.016942	N/A	N/A	-0.642259	0.004074
LDLRAP1	-3.135263	0.006316	N/A	N/A	-0.928326	0.000540
TRAF3IP3	N/A	N/A	-2.791527	0.032925	-0.647528	0.031675
KLF12	-2.125710	0.011243	-2.771125	0.018525	-0.695304	0.012068
LRIG1	-2.738652	0.020579	N/A	N/A	-0.586805	0.013141
NCR3	-2.706076	0.001932	N/A	N/A	-0.633979	0.001441
LY9	-2.019065	0.034464	-2.668549	0.014188	-1.100514	0.000207
MEF2C	-2.624475	0.003700	N/A	N/A	-0.773875	0.041216
UBASH3A	-2.571255	0.011798	N/A	N/A	-0.818329	0.008818
ITPKB	-1.581393	0.010902	-2.509241	0.007590	-0.826938	0.011103
ZBTB20	-2.496229	0.003273	N/A	N/A	-0.772131	0.009490
FCER1A	-2.402110	0.006419	N/A	N/A	-1.552699	0.000579
BCL11B	-0.917419	0.041165	-2.392118	0.035516	-1.474745	0.000174
MBP	-2.377291	0.006302	N/A	N/A	-0.868472	0.007388
CCR3	-2.342655	0.008945	N/A	N/A	-1.572702	0.009451
HLA-DPA1	-2.200394	0.007748	N/A	N/A	-0.936224	0.001776
IL11RA	-2.133219	0.030855	N/A	N/A	-0.930131	0.008327
TAF1C	-2.125968	0.012375	N/A	N/A	-0.874589	0.015083
SBK1	N/A	N/A	-2.072502	0.028802	-1.290117	0.002346
PLEKHG3	-2.066257	0.024804	-1.664436	0.034953	-1.041558	0.023076
EIF3B	N/A	N/A	-2.058015	0.034577	-0.693764	0.002040
LRRN3	-2.046565	0.028636	N/A	N/A	-1.080027	0.000556
SHARPIN	-2.010204	0.008372	N/A	N/A	-0.968491	0.003727
RPS6KA5	-1.967429	0.004629	N/A	N/A	-0.732932	0.013565
FAM159A	N/A	N/A	-1.929378	0.025731	-0.676992	0.004074
IL7R	-1.896507	0.016476	N/A	N/A	-0.790320	0.027966
ZNF395	-1.893392	0.049332	N/A	N/A	-0.787551	0.031747
ABLIM1	-1.853613	0.005466	N/A	N/A	-1.157857	0.009126
DHRS3	-1.843629	0.005709	N/A	N/A	-0.795639	0.000104
CRTAP	N/A	N/A	-1.837619	0.018525	-0.596314	0.009520
CBX7	-1.818787	0.007074	N/A	N/A	-0.725904	0.007580
PLEKHA1	-1.810500	0.014929	N/A	N/A	-1.261706	0.004389
CLYBL	N/A	N/A	-1.797957	0.014039	-0.613287	0.004813
NDE1	-1.787207	0.008093	N/A	N/A	-0.860496	0.004940
FNBP4	-1.783854	0.010042	-1.170237	0.033104	-0.741286	0.042624
MARCKSL1	-1.760652	0.010042	N/A	N/A	-0.648078	0.043650
TCF3	-1.754094	0.032136	N/A	N/A	-0.676168	0.017095
LEF1	-1.723928	0.008673	N/A	N/A	-1.564559	0.000207
TGFBI	-1.711026	0.004309	N/A	N/A	-1.084097	0.044666
LBH	-1.710228	0.006363	N/A	N/A	-0.867130	0.000097
LIME1	-1.690359	0.029884	N/A	N/A	-0.771856	0.004781
SPSB3	-1.656509	0.032354	N/A	N/A	-0.623519	0.010131
LCK	-1.624552	0.006371	N/A	N/A	-0.688850	0.015339
LTBP3	N/A	N/A	-1.590927	0.037013	-0.932992	0.002455

ID3	-1.571687	0.042281	N/A	N/A	-1.085564	0.000034
LAT	-1.555891	0.008093	N/A	N/A	-0.851510	0.005049
CD247	-1.532889	0.034406	N/A	N/A	-0.984938	0.015975
KLHL3	-1.515600	0.025046	N/A	N/A	-0.901367	0.000174
NKTR	-1.497816	0.017694	N/A	N/A	-0.763644	0.019134
WDR74	-1.473994	0.021455	N/A	N/A	-0.717287	0.000952
ITPR3	-1.438716	0.044019	N/A	N/A	-0.608308	0.017043
ZFP36L2	-1.418387	0.010058	N/A	N/A	-0.642430	0.012797
HVCN1	N/A	N/A	-1.406153	0.018018	-0.765061	0.001933
GPR18	-1.399776	0.009274	N/A	N/A	-0.745753	0.031281
CD96	-1.391030	0.042281	N/A	N/A	-1.207798	0.004319
LTB	-1.363133	0.018148	N/A	N/A	-0.821076	0.000526
MORC2	-1.354707	0.009786	N/A	N/A	-0.610719	0.018668
P2RY10	-1.347862	0.013092	N/A	N/A	-0.751282	0.006109
ITK	-1.334017	0.017669	N/A	N/A	-0.693429	0.049987
CCDC92	-1.333479	0.010042	N/A	N/A	-0.825357	0.003726
NFATC2IP	-1.330345	0.033758	N/A	N/A	-0.906281	0.037807
TOMM20	-1.322125	0.013466	N/A	N/A	-0.692099	0.005369
ZNF91	-1.315506	0.007427	N/A	N/A	-0.955420	0.004108
SSBP2	-1.301449	0.047594	N/A	N/A	-0.631617	0.000556
EIF3F	-1.280522	0.013882	N/A	N/A	-0.585987	0.008424
APRT	-1.243239	0.010005	N/A	N/A	-0.594240	0.047373
TAGAP	N/A	N/A	-1.195276	0.048181	-0.655929	0.013273
BTN3A1	-1.187290	0.008693	N/A	N/A	-0.983517	0.015918
OSBPL10	-1.182204	0.038748	N/A	N/A	-0.718950	0.010807
ARHGEF18	-1.152907	0.007376	N/A	N/A	-0.769157	0.001338
FAM43A	N/A	N/A	-1.133956	0.046830	-0.646884	0.003942
PSMF1	-1.115495	0.036333	N/A	N/A	-0.916042	0.009882
MAL	-1.111978	0.018148	N/A	N/A	-1.412302	0.000097
TMEM204	-1.083064	0.013573	N/A	N/A	-1.024654	0.003438
TCF25	-1.053190	0.041530	N/A	N/A	-0.701066	0.003955
CD27	-1.045595	0.042057	N/A	N/A	-0.697795	0.032532
PHACTR4	-1.037980	0.043107	N/A	N/A	-0.630582	0.012883
NMT2	-1.012445	0.018478	N/A	N/A	-0.638898	0.000489
SLC25A38	-1.007351	0.029146	N/A	N/A	-0.619590	0.018179
ST6GAL1	-0.995135	0.015966	N/A	N/A	-0.779525	0.009567
SEC14L1	-0.985257	0.037957	N/A	N/A	-0.993319	0.010072
NPAT	-0.972729	0.024621	N/A	N/A	-0.816373	0.004813
CX3CR1	-0.916116	0.041980	N/A	N/A	-0.927637	0.032532
ADD3	-0.904468	0.037041	N/A	N/A	-0.589061	0.007799
RNF44	-0.903047	0.040853	N/A	N/A	-0.699647	0.005362
BTN3A3	-0.898306	0.025746	N/A	N/A	-0.857395	0.004319
HLA-DPB1	-0.880498	0.032236	N/A	N/A	-0.755800	0.027951
MFNG	-0.878208	0.034110	N/A	N/A	-0.725783	0.004875
RPL22	-0.840123	0.024815	N/A	N/A	-0.639363	0.024750
HLA-DMB	-0.838114	0.021337	N/A	N/A	-0.945217	0.014756
ACVR1	-0.766082	0.038465	N/A	N/A	-0.608937	0.013412
CNNM3	-0.746126	0.038465	N/A	N/A	-0.942659	0.013511
ACP1	-0.735276	0.046443	N/A	N/A	-0.848294	0.033061
SF3A3	-0.693063	0.043760	N/A	N/A	-0.698800	0.045684
OLFM4	6.498703	0.002083	N/A	N/A	2.149982	0.025165
MMP8	4.341540	0.004309	5.802786	0.000177	1.674428	0.009776

LTF	5.253627	0.003440	N/A	N/A	0.819118	0.036932
CD177	4.633437	0.000583	N/A	N/A	1.754744	0.000127
C1QC	N/A	N/A	4.570600	0.011183	1.682468	0.000682
PGLYRP1	4.519776	0.002083	N/A	N/A	1.892541	0.001908
CPD	4.287779	0.000438	N/A	N/A	0.789743	0.002810
IL1R2	4.267536	0.000110	N/A	N/A	3.352011	0.000011
CEACAM8	4.254215	0.004841	N/A	N/A	1.455165	0.012195
MMP9	4.114325	0.000288	N/A	N/A	3.258338	0.000011
VNN1	4.105325	0.000431	N/A	N/A	2.330141	0.000023
GPR84	N/A	N/A	3.857246	0.022079	1.568051	0.002982
CA4	3.814543	0.000560	N/A	N/A	1.608241	0.000150
MPO	3.802001	0.004448	N/A	N/A	1.385744	0.009409
C1QB	3.532817	0.001180	N/A	N/A	2.504405	0.000524
ANKRD22	N/A	N/A	3.487495	0.043298	2.054509	0.002281
DEFA1B	3.471780	0.010475	N/A	N/A	3.287968	0.000540
OLAH	2.412162	0.000911	3.116097	0.045478	0.766092	0.014969
S100A12	3.101817	0.000911	N/A	N/A	3.426841	0.000041
ARG1	3.073698	0.003440	N/A	N/A	2.351097	0.000025
HP	3.060420	0.001408	N/A	N/A	1.773560	0.000903
SOCS3	3.005937	0.008372	N/A	N/A	0.806512	0.018170
ELANE	2.970073	0.033816	N/A	N/A	2.231186	0.003038
ZDHHC19	N/A	N/A	2.956807	0.046074	1.255271	0.002948
EBI3	2.896150	0.023959	N/A	N/A	0.950600	0.017989
IL18R1	2.805586	0.002563	N/A	N/A	1.921909	0.000166
LCN2	2.789121	0.008580	N/A	N/A	1.663780	0.015223
ANXA3	2.780463	0.006367	N/A	N/A	1.903839	0.002346
DEFA4	2.709516	0.030265	N/A	N/A	2.289752	0.008350
BMX	2.683423	0.019660	N/A	N/A	0.675364	0.005598
CD163	2.608475	0.005160	N/A	N/A	2.062995	0.000161
RETN	2.598761	0.003273	N/A	N/A	2.121821	0.000310
CTSG	2.550113	0.024790	N/A	N/A	1.940907	0.008489
PFKFB2	1.724526	0.004374	2.544751	0.003630	0.787344	0.004405
ACSL1	2.496963	0.003511	N/A	N/A	1.558682	0.011159
LILRA3	2.450940	0.002219	N/A	N/A	2.137405	0.001079
C1QA	2.391297	0.002049	N/A	N/A	0.692870	0.016313
MGAM	2.327354	0.008358	N/A	N/A	0.753193	0.017867
TNFAIP6	2.286735	0.014850	N/A	N/A	2.213296	0.004319
CEACAM1	2.283344	0.005539	N/A	N/A	1.760496	0.004116
PADI4	2.188102	0.040948	N/A	N/A	1.238631	0.004641
STOM	2.180863	0.005580	N/A	N/A	1.051036	0.014225
FKBP5	N/A	N/A	2.131990	0.014508	1.564520	0.000005
LAG3	2.119271	0.005466	N/A	N/A	1.553941	0.004387
S100P	2.115748	0.012316	N/A	N/A	2.458790	0.000945
CLEC5A	2.095480	0.005028	N/A	N/A	0.911494	0.001235
CKAP4	2.090271	0.001399	N/A	N/A	1.622436	0.000174
CCRL2	2.066012	0.042281	N/A	N/A	0.767247	0.007444
SMPDL3A	1.968451	0.012602	N/A	N/A	1.297318	0.000524
CTSD	1.968030	0.010042	N/A	N/A	0.745841	0.012883
RGL4	N/A	N/A	1.955828	0.043965	1.456502	0.000879
SAMSN1	1.951153	0.014014	N/A	N/A	1.492716	0.000174
SLC2A3	1.929012	0.005137	N/A	N/A	1.242258	0.002948
HSP90AA1	1.913686	0.005160	N/A	N/A	1.422611	0.002685

IRAK3	1.907424	0.023601	N/A	N/A	1.640127	0.000007
PFKFB3	1.900803	0.006363	N/A	N/A	1.145534	0.014680
CYP1B1	1.900748	0.007848	N/A	N/A	1.492336	0.000148
METTL7B	N/A	N/A	1.896369	0.036865	0.983260	0.002699
ETS2	1.893747	0.001932	N/A	N/A	1.400089	0.000031
SLC39A8	1.857339	0.005491	N/A	N/A	1.386010	0.003471
ADCY3	1.855617	0.005580	N/A	N/A	0.929052	0.024967
ALPL	1.825293	0.036479	N/A	N/A	1.221144	0.042007
HK3	1.783395	0.006637	N/A	N/A	1.532527	0.000161
DHRS9	1.189777	0.036467	1.740812	0.014732	1.142554	0.003220
RNF144B	N/A	N/A	1.736185	0.014732	1.010686	0.011426
LY96	1.732044	0.045233	N/A	N/A	1.164192	0.012883
FKBP4	1.697724	0.033758	N/A	N/A	0.669927	0.041481
S100A9	1.678060	0.007376	N/A	N/A	1.021884	0.000191
TXN	1.670687	0.013765	N/A	N/A	1.316660	0.001933
CLEC1B	1.639792	0.012981	N/A	N/A	1.221570	0.013774
CEACAM6	1.634404	0.029639	N/A	N/A	1.506380	0.005889
HMGB2	1.624286	0.012138	N/A	N/A	1.196928	0.000097
HMOX1	1.618227	0.012566	N/A	N/A	0.686122	0.002271
PSTPIP2	1.616269	0.013882	N/A	N/A	1.140037	0.015897
S100A8	1.615598	0.012676	N/A	N/A	1.854023	0.000041
BCL6	1.476541	0.013180	1.608982	0.032654	1.735913	0.000019
B4GALT5	1.578830	0.008811	N/A	N/A	1.357167	0.000644
BST1	1.573391	0.004448	N/A	N/A	0.612325	0.019198
GYG1	1.534481	0.014359	N/A	N/A	1.639608	0.000031
RPS27L	1.505538	0.041530	N/A	N/A	0.958262	0.034014
GGH	1.490555	0.011838	N/A	N/A	0.695249	0.004120
IL18RAP	1.470914	0.034585	N/A	N/A	1.413553	0.003947
ADM	1.398359	0.024804	N/A	N/A	1.259465	0.006321
FABP5	1.360967	0.036907	N/A	N/A	0.731826	0.001235
UPP1	1.359523	0.039139	N/A	N/A	1.083225	0.000140
ITGAM	1.343670	0.005580	N/A	N/A	0.967217	0.003390
TOP2A	1.338480	0.019639	N/A	N/A	0.742452	0.049051
IER3	1.321341	0.012138	N/A	N/A	0.972304	0.018486
KIF1B	N/A	N/A	1.312971	0.032588	0.794360	0.000277
FCER1G	1.310087	0.011833	N/A	N/A	1.407668	0.000388
VCAN	1.302155	0.034585	N/A	N/A	1.169799	0.004343
LILRB2	1.283732	0.032654	N/A	N/A	0.910341	0.002482
CEBPD	1.265615	0.019259	N/A	N/A	0.887631	0.035485
CNIH4	1.262918	0.042431	N/A	N/A	1.173227	0.001047
NAMPT	1.257692	0.029579	N/A	N/A	1.084812	0.014756
QSOX1	1.245073	0.042681	N/A	N/A	0.686310	0.005125
CD58	1.243478	0.026853	N/A	N/A	0.784745	0.001454
UGCG	1.238123	0.020409	N/A	N/A	0.963021	0.006085
CRISPLD2	1.213744	0.012375	N/A	N/A	0.818943	0.021171
VNN2	1.199885	0.039336	N/A	N/A	0.882664	0.009490
NFKBIA	1.166345	0.034164	N/A	N/A	1.162517	0.017095
MMRN1	1.162756	0.041433	N/A	N/A	0.606909	0.025859
NQO2	1.154300	0.023601	N/A	N/A	0.854849	0.007722
WDFY3	1.121231	0.029267	N/A	N/A	0.616734	0.016873
FGR	1.119998	0.014411	N/A	N/A	0.741998	0.011834
CD63	1.103193	0.015608	N/A	N/A	1.323072	0.000097

RAB27A	1.078784	0.029798	N/A	N/A	0.684018	0.029546
LILRA6	1.065046	0.024804	N/A	N/A	1.318351	0.000646
DRAM1	1.060891	0.033758	N/A	N/A	0.898492	0.003947
DUSP3	1.060373	0.037041	N/A	N/A	0.638469	0.044509
GMFG	1.028847	0.039336	N/A	N/A	0.753195	0.003947
TIMP1	1.015390	0.029540	N/A	N/A	1.352723	0.005889
GNS	1.014337	0.025327	N/A	N/A	0.589806	0.035202
TCN1	1.004216	0.029184	N/A	N/A	1.520543	0.001484
FLOT1	0.994940	0.035393	N/A	N/A	0.975604	0.006663
IFNGR1	0.992835	0.039139	N/A	N/A	1.004609	0.000064
SLA	0.986606	0.049976	N/A	N/A	0.936027	0.000031
FTH1	0.969534	0.035404	N/A	N/A	0.683106	0.024395
TLR8	0.961358	0.032390	N/A	N/A	0.954620	0.001898
RRAGD	0.957908	0.037387	N/A	N/A	0.791835	0.012775
NBN	0.953639	0.022705	N/A	N/A	0.768752	0.022652
TNFAIP3	0.934738	0.048850	N/A	N/A	0.886409	0.041847
TLR5	0.896183	0.035331	N/A	N/A	1.283138	0.002523
PLIN2	0.892200	0.044019	N/A	N/A	0.727577	0.011015
CYBB	0.846435	0.047025	N/A	N/A	0.842549	0.001397
FEN1	0.843919	0.025227	N/A	N/A	0.789386	0.015264
RGL1	0.840981	0.042769	N/A	N/A	0.864149	0.009567
SH3GLB1	0.832503	0.042051	N/A	N/A	0.801403	0.000953
RAB32	0.819004	0.046121	N/A	N/A	0.790021	0.002207
GNA15	0.788169	0.044354	N/A	N/A	0.620528	0.034961
HIST1H2BD	0.784918	0.042328	N/A	N/A	0.989303	0.004133
DDIT4	0.665175	0.047631	N/A	N/A	1.261142	0.000995

Supplementary Figure 1

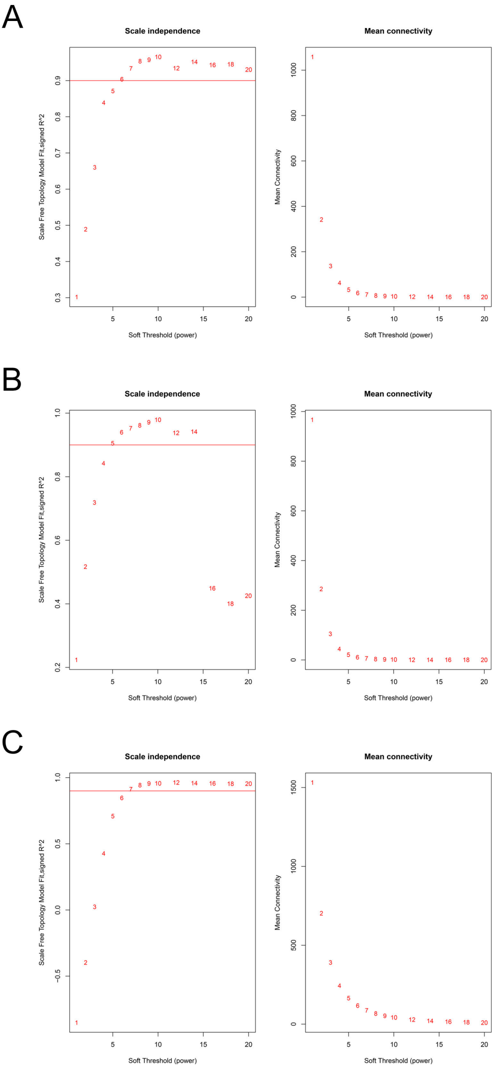

Supplementary Figure 2

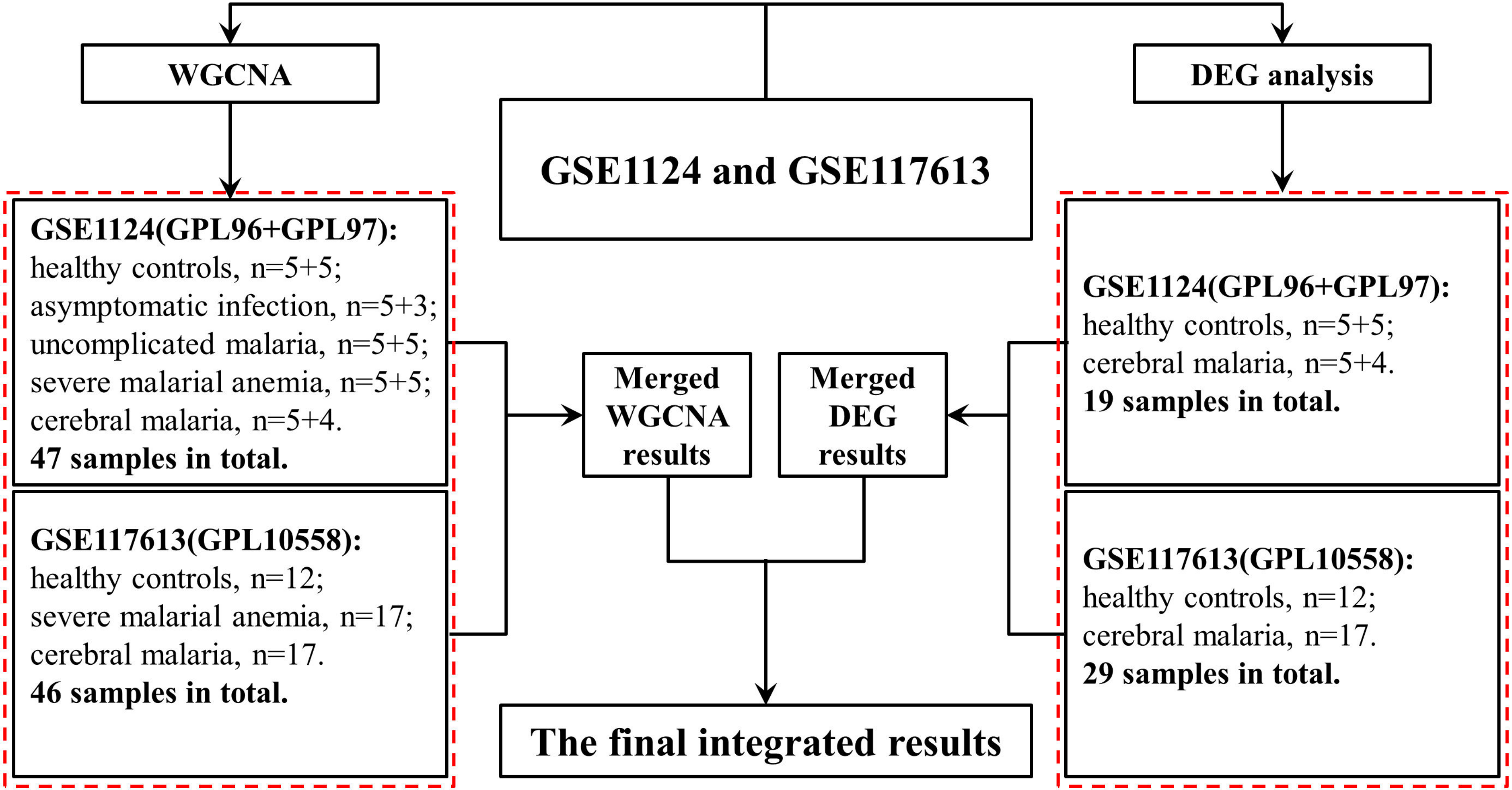

Supplementary Figure 3

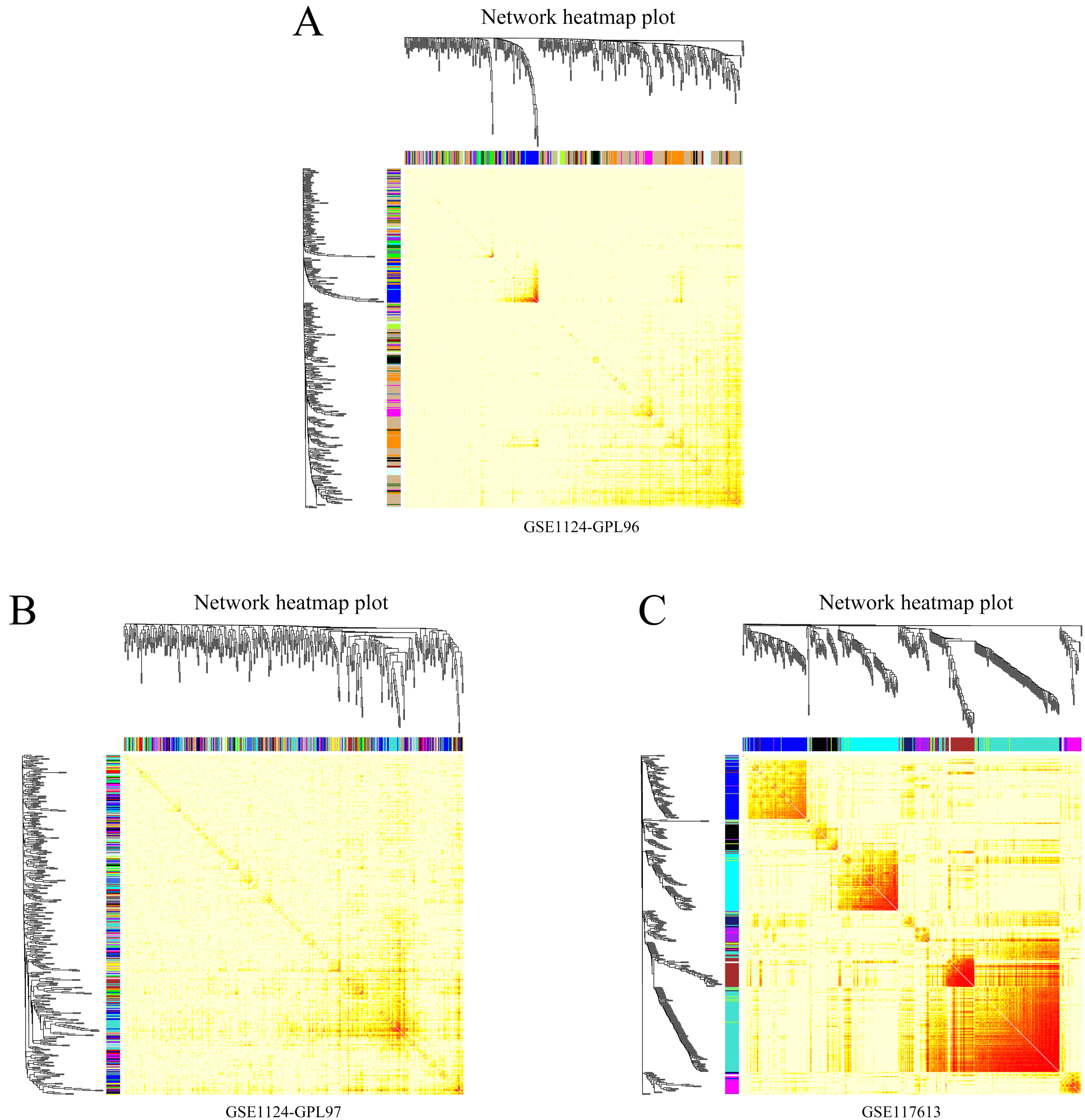

Supplement: Supplementary file 1 — Supporting information. [file IID3-9-819-s001.pdf]
